# Use of Wine and Dairy Yeasts as Single Starter Cultures for Flavor Compound Modification in Fish Sauce Fermentation

**DOI:** 10.3389/fmicb.2019.02300

**Published:** 2019-10-09

**Authors:** Pei Gao, Wenshui Xia, Xinzhi Li, Shaoquan Liu

**Affiliations:** ^1^State Key Laboratory of Food Science and Technology, School of Food Science and Technology, Jiangnan University, Wuxi, China; ^2^Department of Food Science and Technology, National University of Singapore, Singapore, Singapore; ^3^Laboratory of Advanced Food Technology & 3D Printing, National University of Singapore (Suzhou) Research Institute, Suzhou, China

**Keywords:** fish sauce, hydrolysate, tilapia, yeasts, flavor

## Abstract

Effects of wine and dairy yeast fermentation on chemical constituents of tilapia fish head hydrolysate supplemented with glucose in an unsalted and acidic environment were investigated. Three wine yeasts (*Torulaspora delbrueckii* Biodiva, *Saccharomyces cerevisiae* Lalvin EC-1118 and *Pichia kluyveri* Frootzen) and one dairy yeast (*Kluyveromyces marxianus* NCYC1425) were employed as single starter cultures, respectively, and were compared with one soy sauce yeast (*Candida versatilis* NCYC1433). Each yeast showed different growth kinetics and fermentation performance. Compared with *C. versatilis* NCYC1433, other yeasts presented a significant higher rate of glucose consumption (*P* < 0.05). Besides, *K. marxianus* NCYC1425 and *P. kluyveri* Frootzen produced more citric acid and succinic acid, respectively, while *S. cerevisiae* Lalvin EC-1118 exhibited higher pyruvic acid production. Significant lower levels of total free amino acids were observed in samples inoculated with wine yeasts relative to other yeasts (*P* < 0.05). Non-soy sauce yeasts produced increased various levels of esters and alcohols without traditional fish sauce unpleasant odorants, especially *K. marxianus* NCYC1425 and *P. kluyveri* Frootzen. The results confirmed that non-soy sauce yeasts are suitable for fish sauce flavor compound modification and to develop a fast fermentation process for saltless fish sauce from fish head, which could increase the acceptability of fish sauce and improve the utilization of fish by-products.

## Introduction

Fish are an extremely important source of protein. In 2015, fish accounted for about 17 percent of global human animal protein consumption, and global fish production peaked at about 171 million tonnes in 2016 (FAO, 2018). However, over 50 percent (w/w) of the weight of fish as by-products are generated accompanying the processing chain (Kristinsson and Rasco, 2000), including heads, tails, bones, skins, scales and intestines. At present, the industrial fish by-products are normally used for the manufacture of low-value products such as animal feed, or even directly discarded as wastes (Kristinsson and Rasco, 2000). These treatments are not only a huge waste of protein resources, but also a pollution of the environment if not treated before disposal. These problems greatly limit the development and utilization of fish by-products.

Fish sauce is a type of fermented liquid condiments usually made from low-value fish in Southeast and East Asian countries (Lopetcharat et al., 2001). Upon widespread recognition of its ability to impart a savory (umami) taste to dishes, fish sauce has become more accepted globally. A series of physical, biological and chemical reactions occur during the fermentation of fish sauce, including oxidative rancidity of unsaturated fatty acids and the action of exogenous enzymes and endogenous microorganisms (Lopetcharat et al., 2001). As a result, fish sauce is prone to producing undesirable flavor substances such as medium-chain aldehydes, branched-chain carboxylic acids, volatile nitrogen and sulfur-containing compounds (Fukami et al., 2002, 2004; Jiang et al., 2008; Giri et al., 2010; Yoshikawa et al., 2010). These compounds impart distinct fishy, sweaty, rancid and fecal notes to fish sauce, which seriously limit its acceptability. Therefore, there is a pressing need to modify fish sauce flavor so as to attract more consumers.

The usage of microbial inoculation to enhance flavor has become a common practice in fermentation. *Zygosaccharomyces rouxii*, *Candida versatilis or Candida etchellsii* and *Pichia guilliermondii*, isolated from traditional soy sauce, have been effectively employed as starter cultures to enhance the flavor of soy sauce individually or in co-culture with lactic acid bacteria (LAB) (Harada et al., 2017; Singracha et al., 2017; Devanthi et al., 2018). Yoshikawa et al. (2010) reported that inoculation of *Z. rouxii* and *C. versatilis* NCYC1433 coupled with koji and *Tetragenococcus halophilus* was effective for conferring the soy-sauce-like flavor and increasing free amino acids and ethanol contents in Chum salmon sauce. To a certain extent, the application of yeasts could reduce the salt level due to production of inhibitory substances such as alcohols (Singracha et al., 2017). But a considerable salt concentration is still essential for the survival of these soy sauce yeasts, which poses health risks to consumers because of high salt levels. Thus, the use of non-halophilic yeasts to reduce or even eliminate salt addition is of paramount importance. This is the first report on employment of non-soy sauce yeasts for the fermentation of a novel fish sauce from fish head hydrolysate.

In this study, three species of wine yeasts (*Torulaspora delbrueckii*, *Saccharomyces cerevisiae* and *Pichia kluyveri*) and one species of dairy yeast (*Kluyveromyces marxianus*) with a high aromatic capacity were employed as single starter cultures for fish sauce fermentation, in comparison to one species of soy sauce yeast (*C. versatilis*). The aim was to ascertain whether non-soy sauce yeasts were suitable for fish sauce flavor modification and to develop a fast fermentation process for saltless fish sauce with better flavor.

## Materials and Methods

### Materials

Nile tilapia (*Oreochromis niloticus*) heads used in this experiment were prepared in the laboratory from tilapia whole fish that was purchased from a local market (Singapore). Flavourzyme (a fungal protease/peptidase complex) from *Aspergillus oryzae* (≥ 500 U/g), was supplied by Sigma-Aldrich Pte Ltd (Saint Louis, MO, United States). *T. delbrueckii* Biodiva and *S. cerevisiae* subsp. *bayanus* Lalvin EC-1118 were purchased from Lallemand (Brooklyn Park, Australia). *P. kluyveri* FrootZen was bought from Chr. Hansen (Hørsholm, Denmark). *K. marxianus* NCYC 1425 and *C. versatilis* NCYC 1433 were obtained from National Collection of Yeast Cultures (Norwich, England). D-Glucose standard and amino acid standards were purchased from Sigma (Saint Louis, MO, United States). Other chemicals used in this study were of analytical grade.

### Preparation of Yeast Starter Cultures

Prior to inoculation, five yeast strains were respectively transferred from the content of a 25% glycerine-water solution to YM broth, containing 0.3% (w/v) yeast extract, 0.3% (w/v) malt extract, 0.5% (w/v) peptone and 1% (w/v) glucose. After cultivating at 30°C for 24h, yeast cell pellets were harvested by centrifugation (Centrifuge 5810R, Hamburg, Germany) at 12,000 rpm for 10 min at 4°C. Then these cell pellets were washed twice with an equivalent volume of 0.85% saline (w/v), and re-suspended therein as the starter cultures.

### Production of Fish Hydrolysate and Sauce

**FIGURE 1:**
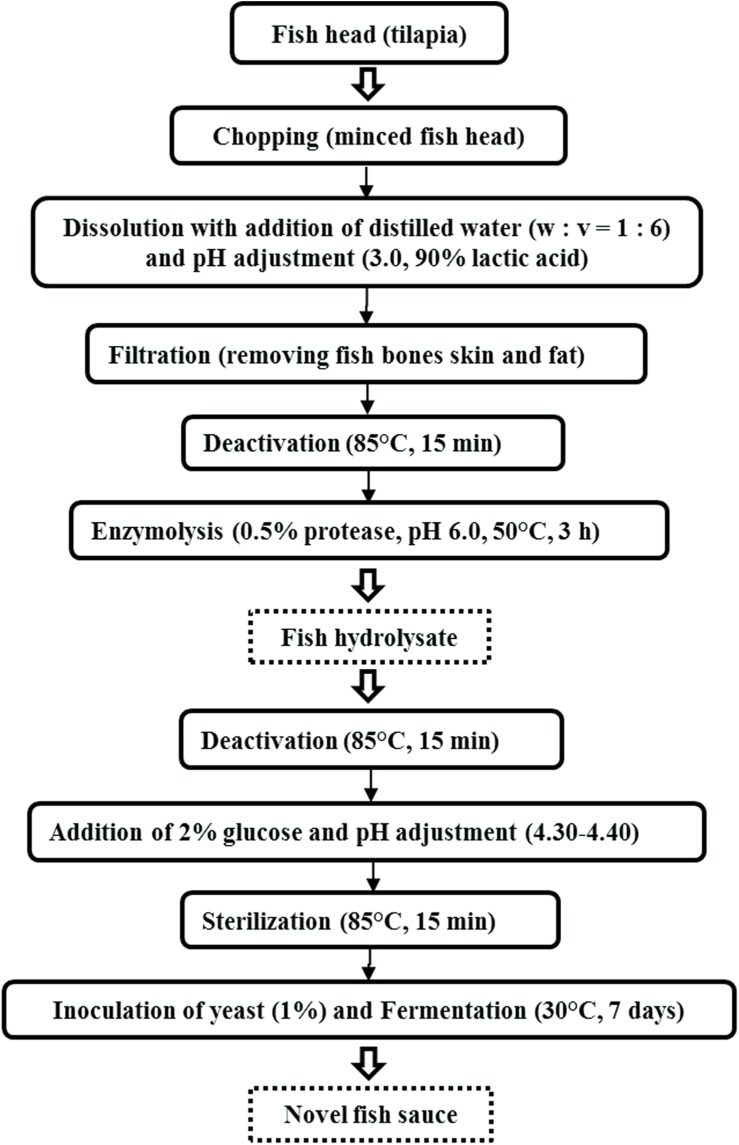
The process for the production of fish hydrolysate and sauce.

The process for the production of fish hydrolysate and sauce is shown in Figure 1. Fish heads were removed from the freezer (−20°C) to thaw and crushed with a 400 W Philips HR-1396 Viva Collection Chopper (Amsterdam, Holland). Distilled water was added into the minced fish head (w: v = 1: 6), and the pH of the mixture was adjusted to 3.0 using 90% lactic acid. This was followed by filtering through a gauze to remove insoluble fish bones and skin. Then the filtrate was placed in a 4°C fridge overnight to remove the fat floating on top of the filtrate. The filtrate was subsequently heated in an 85°C-water bath for 15 min to deactivate the fish endogenous enzymes.

Thereafter, pH of the filtrate was shifted to 6.0 by adding 1 M NaOH, and kept in an 50°C-oscillating bath (Julabo SW22, Seelbach, Germany) added with 0.5% (v/v) protease at a shaking speed of 120 rpm (degree of hydrolysis reached 35–40%). After 3 h, the hydrolysate was heated at 85°C for 15 min to deactivate the protease. Next, the hydrolysate was added with 2% (w/v) glucose and the pH was shifted to 4.30–4.40 by adding 90% lactic acid, and sterilized in a Hirayama HV-50 Upright Autoclave (Saitama, Japan) at 85°C (Lopetcharat et al., 2001) for 15 min. Heat treatment was effective for microbes elimination, verified by plating on potato dextrose agar (PDA).

Finally, the cooled sterilized hydrolysate was divided into five portions and each portion was inoculated with the above yeast starter cultures respectively, including S1 (*K. marxianus* NCYC1425), S2 (*T. delbrueckii* Biodiva), S3 (*S. cerevisiae* Lalvin EC-1118), S4 (*P. kluyveri* Frootzen), and S5 (*C. versatilis* NCYC1433). These portions were incubated statically at 30°C for 7 days, and fermentation was conducted in triplicate for each yeast.

Samples were collected at day 0, 1, 3, 5 and 7, and analyzed for yeast enumeration, pH and chemical constituents. The pH was measured directly with a pH meter (Metrohm, Herisau, Switzerland). Yeasts were enumerated using the method of spread plating on PDA medium at 30°C for 48 h. The results were expressed as log CFU/mL. Each sample was evaluated in triplicate.

### Non-volatile Constituents Analysis

Sugar (glucose) and seven organic acids (citric acid, pyruvic acid, malic acid, succinic acid, lactic acid, and acetic acid) were analyzed according to the method of Lu et al. (2018) using a Shimadzu HPLC. The mobile phase was acetonitrile and water (v: v = 80: 20), and 0.1% (v/v) sulfuric acid, respectively. The results were expressed as g/L. Each sample was evaluated in triplicate.

Analysis of free amino acids was carried out using the ARACUS Amino Acid Analyzer (MembraPure GmbH, German), connected to a column composed of lithium system (125 mm BL × 4.0 mm ID) and a ammonium absorber (40 mm × 2.0 mm ID). Eluent A to Eluent F were used at the flow rate of 240 μL/min. The column temperature and the reactor temperature was 50 and 130°C, respectively. Quantification was performed by using a 100 nmol/mL external standard solution including 18 amino acids or derivatives (except Cys, 50 nmol/mL). The results were expressed as mg/L. Each sample was evaluated in triplicate.

### Volatile Constituents Analysis

Volatile compounds of fish sauce were extracted by using the method of head space solid-phase microextraction (HS-SPME) as reported previously (Gao et al., 2016a) with some modification. Each sample was heat treated at 60°C for 5 min before extraction. A CAR/PDMS fiber (85 μm, Supelco, Bellefonte, PA, United States) was then inserted into the headspace of the sample vial.

Agilent 7890A GC with Agilent 5795C inert MSD with Triple-Axis Detector was used for GC-MS analysis. It was carried out according to the previous method (Gao et al., 2016a). The volatile flavor compounds were identified according to NIST 2014 standard library and Kovats index (KI), and 2-octanol (164.4 μg/L) was used as an internal standard. The concentration of volatile compounds was calculated by comparing the peak area of each compound with that of the internal standard, expressed as mg/L. Each sample was evaluated in triplicate.

### Statistical Analysis

The experiments were designed and analyzed statistically by ANOVA, and mean value differences were evaluated by using Duncan’s multiple range test (*P* < 0.05). Statistical analysis was performed using the SPSS statistic program (Version 19.0, SPSS Inc., Chicago, IL).

## Results

### Yeast Populations and pH

**FIGURE 2:**
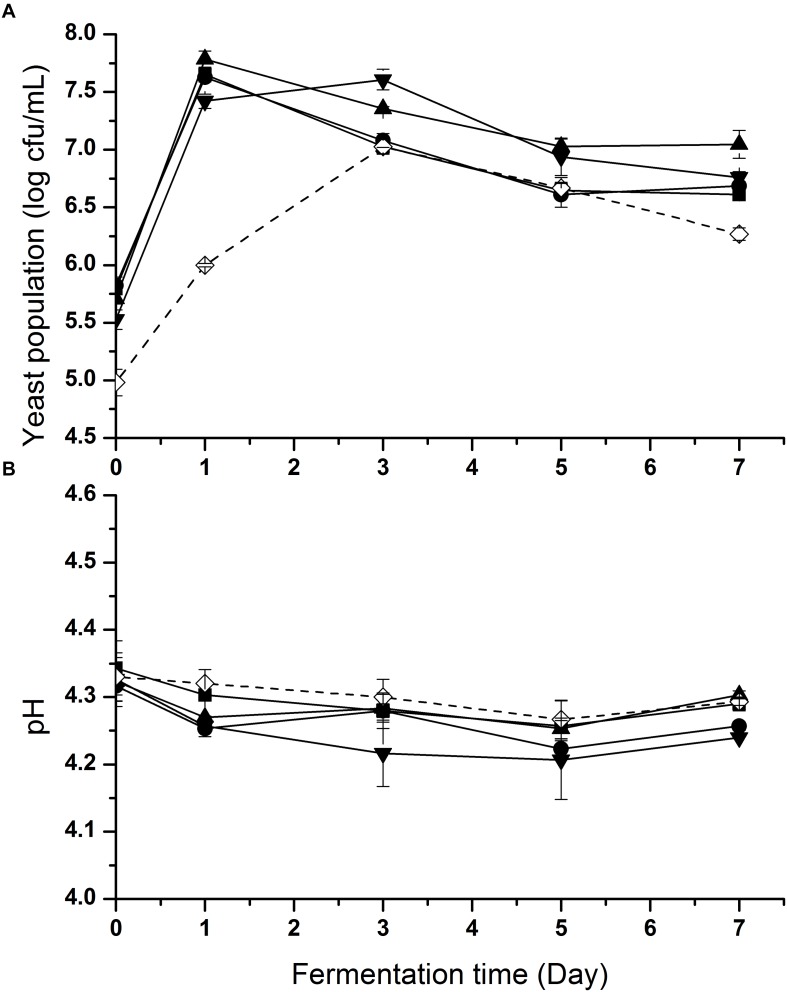
Yeast population **(A)** and pH **(B)** changes of fish sauce during fermentation with different yeasts. S1 (■): inoculated with *Kluyveromyces marxianus* NCYC1425; S2 (●): inoculated with *Torulaspora delbrueckii* Biodiva; S3 (▲): inoculated with *Saccharomyces cerevisiae* Lalvin EC-1118; S4 (▼): inoculated with *Pichia kluyveri* Frootzen; S5 (◊): inoculated with *Candida versatilis* NCYC1433.

In order to accelerate the growth of yeasts, 2% (w/v) glucose was added to each inoculated sample. Changes of yeast cell populations in inoculated samples during fermentation are shown in Figure 2A. Yeast populations in all samples presented a first increasing and then decreasing trend. An increase of around 2 log CFU/mL was observed in each inoculated sample. In S1-S4 (wine and dairy yeasts), the yeast counts reached the level of about 7.5 log CFU/mL after day 1 of fermentation, while on day 3 S5 (soy sauce yeast) reached 7.0 log CFU/mL.

In order to inhibit the growth of pathogenic and spoilage bacteria, the environment of the hydrolysate was adjusted to acidic conditions before fermentation in the absence of LAB. The pH changes of all samples during fermentation are shown in Figure 2B. At the beginning, the pH of all samples ranged from 4.3 to 4.4. A slight decreasing trend was observed in all samples during fermentation as a whole until day 5, when pH began to rise slightly.

### Sugar and Organic Acids

**FIGURE 3:**
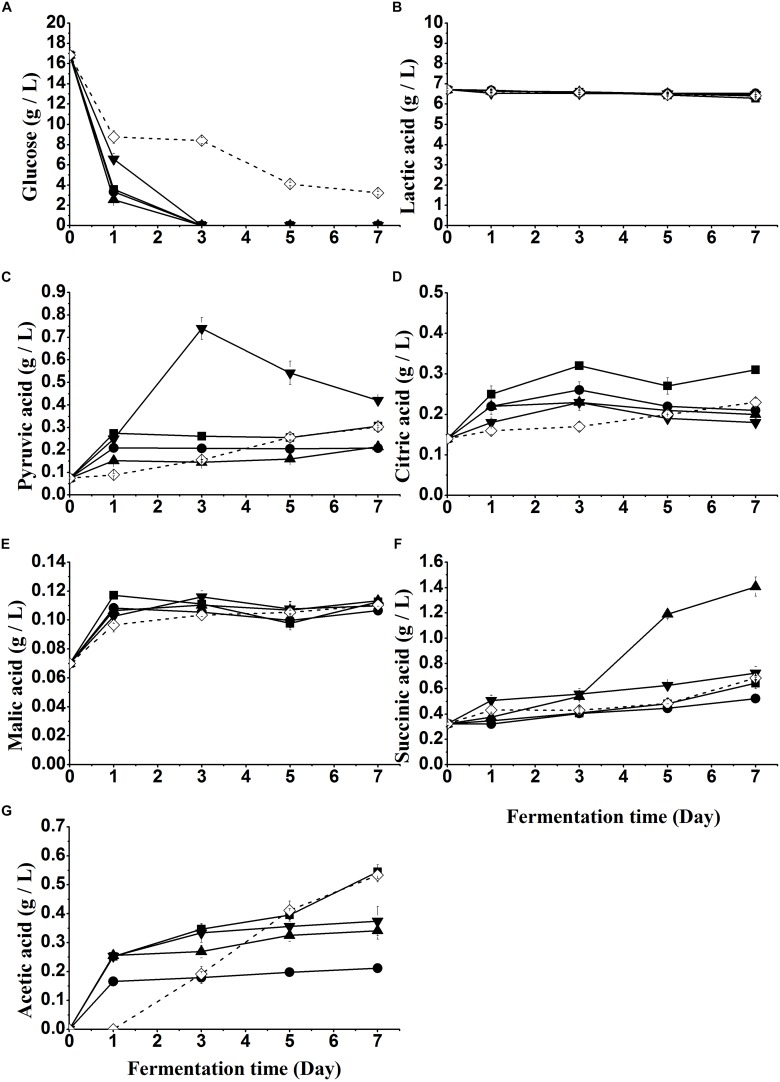
Glucose **(A)** and organic acids **(B–F)** changes of fish sauce during fermentation with different yeasts. S1 (■): inoculated with *Kluyveromyces marxianus* NCYC1425; S2 (●): inoculated with *Torulaspora delbrueckii* Biodiva; S3 (▲): inoculated with *Saccharomyces cerevisiae* Lalvin EC-1118; S4 (▼): inoculated with *Pichia kluyveri* Frootzen; S5 (◊): inoculated with *Candida versatilis* NCYC1433.

Sugar (glucose) changes in all samples with yeast treatment are shown in Figure 3A. A significant (*P* < 0.05) decline was observed on day 1 of fermentation in each sample, during which yeast counts of S1-S4 reached the maximum of 7.5 log CFU/mL (Figure 2A). No glucose was detected in S1-S4 from day 3, while glucose decreased continuously with a residual content of 3.21 g/L on day 7 in S5.

It is well known that organic acids are involved in yeast fermentation. As can be seen, no significant differences were observed in the concentration of lactic acid (Figure 3B) while that of other organic acids significantly increased after the fermentation of yeasts, leading to a slight decrease of pH. The difference was ascribed to sugar metabolism by these yeasts to form “other organic acids” but no or little lactic acid, which is usually produced by LAB through lactic acid fermentation (Lu et al., 2017a). In addition, due to the usage of lactic acid for the acidity adjustment, lactic acid exhibited the highest concentration at the beginning.

Although *P. kluyveri* Frootzen had a lower rate of glucose consumption (Figure 3A), more pyruvic acid (Figure 3C) was produced by S4 (*P. kluyveri* Frootzen) within the first 3 days, than others including S3 (*S. cerevisiae*). Citric acid (Figure 3D), malic acid (Figure 3E) and succinic acid (Figure 3F) are known to be the critical intermediate products of the TCA cycle. The three acids significantly increased at the early stage of fermentation. At the later stage, malic acid remained stable and citric acid slightly reduced, while succinic acid kept increasing with a faster rate in S3 (*S. cerevisiae*). A significantly (*P* < 0.05) higher level of acetic acid (Figure 3G) was observed in samples inoculated with *K. marxianus* NCYC1425 (S1) and *C. versatilis* NCYC1433 (S5), indicating more acetaldehyde was metabolized in S1 and S5 to generate acetic acid. This was also consistent with the result that non-*Saccharomyces* yeasts were usually high producers of acetic acid reported by Contreras et al. (2014).

### Free Amino Acids

The concentration and composition of free amino acids (FAA) in fish hydrolysate and fish sauce are shown in Table 1. After the flavourzyme treatment, 17 FAA were detected in unfermented fish hydrolysate with concentration of 5296 mg/L. A extremely low concentration (4 mg/L) was observed in cysteine. A similar result was reported by Robert et al. (2015) who found the lowest amount of cysteine (FFA, 0.04%) in the by-products (head, frames and viscera) hydrolysate from the same species of Tilapia.

**TABLE 1:** Free amino acids (FAA) in fish sauce fermented with different yeasts (mg/L).

**FAA**	**Taste**	**Fish hydrolysate**	**Fish sauce (Day 7)**				
			
			**S1**	**S2**	**S3**	**S4**	**S5**
Asp	Umami	228.93 ± 10.66^ab^	228.00 ± 6.17^ab^	271.30 ± 65.84^a^	204.00 ± 14.79^b^	249.92 ± 8.50^ab^	218.86 ± 2.95^ab^
Thr	Sweet	196.15 ± 14.42^ab^	164.94 ± 2.71^b^	168.00 ± 40.91^b^	100.34 ± 4.18^c^	209.85 ± 5.02^a^	212.39 ± 1.26^a^
Ser	Sweet	158.23 ± 18.08^a^	144.88 ± 8.30^ab^	147.55 ± 20.10^ab^	115.92 ± 20.91^b^	172.31 ± 7.07^a^	170.53 ± 3.86^a^
Glu	Umami	526.28 ± 37.23^abc^	487.69 ± 28.53^cd^	544.82 ± 11.72^ab^	466.11 ± 21.83^d^	544.97 ± 3.52^a^	498.82 ± 16.85^bcd^
Pro	Sweet	1089.59 ± 12.20^c^	1330.52 ± 221.36^b^	481.47 ± 7.14^d^	1587.64 ± 9.21^a^	1563.47 ± 13.82^a^	1661.33 ± 24.18^a^
Gly	Sweet	131.27 ± 7.45^a^	140.08 ± 5.76^a^	142.73 ± 13.32^a^	135.23 ± 1.93^a^	142.78 ± 2.84^a^	135.43 ± 4.89^a^
Ala	Sweet	227.48 ± 8.64^c^	268.07 ± 15.25^bc^	278.02 ± 7.62^b^	258.60 ± 26.42^bc^	338.87 ± 21.81^a^	258.63 ± 26.05^bc^
Cys	—	4.19 ± 0.39^a^	3.77 ± 0.22^ab^	2.59 ± 0.24^c^	3.10 ± 0.30^bc^	3.39 ± 0.62^b^	3.40 ± 0.33^b^
Val	—	307.87 ± 4.80^a^	281.59 ± 2.22^b^	307.36 ± 14.44^a^	284.36 ± 15.08^b^	282.29 ± 12.97^b^	291.59 ± 14.58^ab^
Met	Bitter	169.30 ± 6.55^a^	137.67 ± 2.12^c^	145.43 ± 7.21^bc^	139.81 ± 18.24^c^	158.81 ± 7.35^ab^	162.14 ± 7.30^ab^
Ile	Bitter	222.73 ± 8.01^a^	181.58 ± 5.85^c^	211.84 ± 5.12^ab^	177.34 ± 14.37^c^	193.30 ± 21.02^bc^	226.40 ± 3.03^a^
Leu	Bitter	504.61 ± 19.55^a^	414.10 ± 8.16^cd^	460.49 ± 8.20^abc^	372.61 ± 26.31^d^	425.52 ± 51.87^bc^	471.47 ± 20.33^ab^
Tyr	Bitter	275.95 ± 10.51^ab^	259.58 ± 7.10^b^	269.13 ± 15.07^ab^	269.31 ± 14.49^ab^	254.15 ± 18.53^b^	305.61 ± 29.92^a^
Phe	Bitter	399.21 ± 19.16^bc^	372.67 ± 14.91^c^	410.99 ± 25.65^b^	384.12 ± 30.51^bc^	384.51 ± 6.34^bc^	460.22 ± 13.20^a^
His	Bitter	118.59 ± 5.72^a^	106.72 ± 2.22^ab^	113.68 ± 5.40^ab^	104.33 ± 11.00^b^	117.39 ± 3.63^a^	116.55 ± 2.76^a^
Lys	Bitter	406.65 ± 38.16^ab^	423.41 ± 16.72^b^	390.52 ± 29.75^b^	382.34 ± 8.47^b^	451.82 ± 9.37^a^	450.22 ± 3.72^a^
Arg	Bitter	328.95 ± 15.87^a^	309.44 ± 9.26^ab^	311.35 ± 23.94^ab^	298.00 ± 2.97^b^	310.37 ± 10.38^ab^	322.73 ± 3.57^ab^
Bitter		2426.00 ± 104.38^ab^	2205.18 ± 58.15^c^	2313.44 ± 58.82^b^	2127.87 ± 31.70^c^	2295.86 ± 74.53^b^	2515.34 ± 25.13^a^
Umami		755.22 ± 43.99^ab^	715.69 ± 33.85^bc^	816.12 ± 77.51^a^	670.11 ± 34.67^c^	794.89 ± 5.25^ab^	717.68 ± 19.69^bc^
Sweet		1802.72 ± 41.28^c^	2048.50 ± 239.37^b^	1217.77 ± 54.81^d^	2197.73 ± 42.71^b^	2427.28 ± 21.68^a^	2438.30 ± 15.39^a^
Total		5296.00 ± 171.61^b^	5254.73 ± 321.37^b^	4657.29 ± 115.05^d^	5283.16 ± 51.40^b^	5803.72 ± 102.00^a^	5966.30 ± 23.43^a^

*S1, inoculated with *Kluyveromyces marxianus* NCYC1425; S2, inoculated with *Torulaspora delbrueckii* Biodiva; S3, inoculated with *Saccharomyces cerevisiae* Lalvin EC-1118; S4, inoculated with *Pichia kluyveri* Frootzen; S5, inoculated with *Candida versatilis* NCYC1433. ^*a*–*d*^Values in the same row with different letters were significantly different (*P* < 0.05). Results: Mean ± SD, *n* = 3.*

The total FAA of S4 and S5 (5804 mg/L and 5966 mg/L, respectively) was significantly (*P* < 0.05) increased compared with fish hydrolysate, indicating that *P. kluyveri* Frootzen and *C. versatilis* NCYC1433 had a relatively stronger ability of proteolysis to form amino acids. Conversely, the significant (*P* < 0.05) decline of total FAA in S2 (*T. delbrueckii*, 4657 mg/L) indicated a higher capacity of *T. delbrueckii* Biodiva to metabolize amino acids. Moreover, all samples except S2 presented more sweet amino acids, among which S1 and S3 presented less bitter amino acids at the same time. The concentration of umami amino acids significantly (*P* < 0.05) decreased in S3, while no significant differences were observed in the other inoculated samples relative to the fish hydrolysate (*P* > 0.05). Individually, significantly lower amounts of threonine, proline, isoleucine and leucine in S1, threonine and proline in S2, threonine, serine, isoleucine and leucine in S3, isoleucine in S4 were observed compared with S5 (*P* < 0.05). Inversely, S4 displayed a higher level of alanine than S5 (*P* < 0.05). In addition, a slight decrease in methionine was found in inoculated samples compared with the starting matrix.

### Volatile Compounds

The fish hydrolysate and fish sauce fermented with different yeasts were analyzed for the volatile compounds (Table 2). The main odor components of fish hydrolysate were aldehydes and furans, accounting for 59% and 22% of total content respectively. Compared with 34 volatiles before fermentation, 47, 47, 46, 38, and 32 volatiles were detected in S1-S4 (wine and dairy yeasts) and S5 (soy sauce yeast) at the end of fermentation, respectively. And individual concentration of S1–S5 was 2512, 1323, 1590, 8962, and 848 μg/L, respectively, compared to 1142 μg/L of fish hydrolysate. The result indicated that wine yeast and dairy yeast could improve the flavor of fish sauce quantitatively compared with soy sauce yeast, of which *P. kluyveri* Frootzen displayed the maximum improvement.

**TABLE 2:** Volatile compounds in fish sauce fermented with different yeasts (μg/L).

**RT**	**KI**	**Compound name**	**Indentification**	**Fish hydrolysate**	**Fish sauce (Day 7)**					**OT (μg/L)**
						
					**S1**	**S2**	**S3**	**S4**	**S5**	
6.08	-	Ethyl acetate	MS	ND	687.93 ± 32.40b	31.17 ± 2.69c	30.56 ± 5.39c	3536.32 ± 651.58a	2.75 ± 0.37c	5^a^
7.34	944	Propanoic acid, ethyl ester	MS,KI	ND	7.90 ± 1.47	ND	ND	ND	ND	NF
8.55	1009	Isobutyl acetate	MS,KI	ND	ND	ND	ND	22.60 ± 9.83	ND	25^a^
10.05	1067	Acetic acid, butyl ester	MS,KI	ND	ND	ND	ND	5.89 ± 0.61	ND	58^a^
11.30	1114	1-Butanol, 3- methyl-, acetate	MS,KI	ND	21.67 ± 0.99b	ND	12.06 ± 3.86b	1172.36 ± 57.44a	ND	1.6^a^
12.67	1157	Acetic acid, pentyl ester	MS,KI	ND	4.80 ± 0.24b	ND	ND	47.23 ± 12.10a	ND	43^b^
15.30	1265	Acetic acid, hexyl ester	MS,KI	ND	51.94 ± 1.83b	0.46 ± 0.12c	22.24 ± 4.36c	419.26 ± 34.31a	ND	115^c^
16.51	1318	n-Caproic acid vinyl ester	MS,KI	39.66 ± 1.01a	-	1.96 ± 0.19b	2.20 ± 0.32b	ND	ND	NF
16.64	1325	Heptanoic acid, ethyl ester	MS,KI	ND	1.02 ± 0.08c	1.47 ± 0.05b	2.38 ± 0.41a	ND	ND	2^a^
17.20	1353	Ethyl lactate	MS,KI	ND	18.87 ± 0.56ab	15.83 ± 1.03c	17.71 ± 2.44bc	ND	20.91 ± 1.47a	NF
17.47	1366	Acetic acid, heptyl ester	MS,KI	ND	9.26 ± 0.92b	ND	ND	72.96 ± 1.31a	ND	NF
17.63	1374	Acetic acid, 2-ethylhexyl ester	MS,KI	ND	ND	ND	ND	7.52 ± 2.50	ND	NF
18.27	1407	3-Hepten-1-ol, acetate	MS,KI	ND	ND	ND	0.58 ± 0.08b	9.44 ± 0.68a	ND	NF
18.58	1427	Octanoic acid, ethyl ester	MS,KI	ND	8.12 ± 0.68c	14.09 ± 1.63b	ND	19.17 ± 3.44a	ND	19.4^a^
19.28	1469	Acetic acid, octyl ester	MS,KI	ND	6.11 ± 0.60bc	ND	11.01 ± 1.14b	101.96 ± 10.42a	ND	5000^d^
19.84	1503	3-Octen-1-ol, acetate, (Z)-	MS,KI	ND	ND	ND	ND	5.27 ± 0.45	ND	NF
19.95	1510	2,4-Hexadienoic acid, ethyl ester	MS,KI	ND	8.94 ± 0.80a	3.48 ± 0.19b	3.34 ± 0.23b	3.06 ± 0.22b	ND	NF
20.23	1530	Non-anoic acid, ethyl ester	MS,KI	ND	10.55 ± 1.55a	11.82 ± 1.67a	9.98 ± 0.73a	10.37 ± 0.54a	ND	377^e^
20.85	1573	Acetic acid, nonyl ester	MS,KI	ND	1.99 ± 0.29b	ND	8.57 ± 0.43b	70.70 ± 8.74a	ND	NF
20.97	1581	3-Methylbutyl 2-methoxyacetate	MS,KI	ND	7.13 ± 0.77a	ND	2.19 ± 0.45b	ND	ND	NF
21.67	1634	Decanoic acid, ethyl ester	MS,KI	ND	6.70 ± 1.27d	28.39 ± 8.09b	149.77 ± 1.99a	18.13 ± 2.80c	ND	5^a^
21.96	1656	Octanoic acid, 3-methylbutyl ester	MS,KI	ND	ND	ND	4.56 ± 0.31	ND	ND	NF
22.02	1661	4-Decenoic acid, ethyl ester, (Z)-	MS,KI	ND	1.49 ± 0.28a	1.74 ± 0.34a	1.40 ± 0.21a	1.94 ± 0.39a	ND	NF
22.21	1676	Acetic acid, decyl ester	MS,KI	ND	ND	ND	1.06 ± 0.18b	5.43 ± 0.57a	ND	NF
22.36	1687	Ethyl 9-decenoate	MS,KI	ND	ND	0.98 ± 0.02b	4.49 ± 0.25a	ND	ND	NF
22.57	1704	3-Decen-1-ol, acetate, (Z)-	MS,KI	ND	ND	ND	3.10 ± 0.21b	4.66 ± 0.55a	ND	NF
23.04	1744	Acetic acid, phenylmethyl ester	MS,KI	ND	ND	ND	ND	4.53 ± 1.18	ND	NF
24.08	1834	Acetic acid, 2-phenylethyl ester	MS,KI	8.41 ± 3.34c	253.50 ± 21.41b	3.69 ± 2.60c	4.50 ± 0.51c	1074.26 ± 103.84a	ND	249.6^a^
24.15	1841	Dodecanoic acid, ethyl ester	MS,KI	ND	3.12 ± 0.52b	2.60 ± 0.66b	24.67 ± 0.65a	3.62 ± 0.88b	ND	NF
24.82	1902	Propanoic acid, 2-phenylethyl ester	MS,KI	ND	8.38 ± 1.27	ND	ND	ND	ND	NF
26.32	2049	Tetradecanoic acid, ethyl ester	MS,KI	ND	ND	ND	1.50 ± 0.11	ND	ND	NF
		**Subtotal**		48.07 ± 3.43e	1119.43 ± 60.54b	119.31 ± 9.21d	317.70 ± 14.22c	6620.74 ± 814.00a	23.66 ± 1.66e	
19.06	1455	Acetic acid	MS,KI	ND	40.14 ± 3.08b	16.06 ± 2.40c	22.95 ± 9.07c	66.96 ± 14.03a	25.48 ± 1.48c	5.5^a^
24.19	1844	Hexanoic acid	MS,KI	13.94 ± 0.57b	ND	12.55 ± 1.86b	ND	16.88 ± 1.39a	13.97 ± 0.84b	3000^e^
24.99	1918	4-Hexenoic acid	MS,KI	ND	10.71 ± 0.46a	6.60 ± 0.66b	5.67 ± 0.91b	ND	ND	NF
25.33	1951	Heptanoic acid	MS,KI	ND	ND	ND	1.92 ± 0.36a	ND	0.99 ± 0.11b	3000^e^
26.41	2058	Octanoic acid	MS,KI	2.12 ± 0.23c	2.97 ± 0.24c	5.89 ± 1.52c	50.53 ± 2.62a	35.76 ± 8.67b	1.52 ± 0.63c	3000^e^
27.39	2158	Sorbic acid	MS,KI	14.30 ± 1.21a	2.67 ± 0.23	3.35 ± 0.82	1.68 ± 0.29	11.42 ± 2.57b	2.57 ± 0.76c	NF
27.45	2164	Non-anoic acid	MS,KI	6.46 ± 1.39a	5.87 ± 0.51ab	4.32 ± 1.55bc	3.75 ± 0.20c	7.53 ± 0.82a	1.68 ± 0.17d	3000^e^
28.52	2270	Decanoic acid	MS,KI	ND	ND	5.16 ± 1.77c	24.23 ± 0.49a	7.55 ± 1.45b	0.90 ± 0.51d	10000^e^
31.11	2483	Dodecanoic acid	MS,KI	ND	ND	ND	2.65 ± 0.26	ND	ND	NF
		**Subtotal**		36.82 ± 0.49d	62.36 ± 3.18c	53.92 ± 8.52cd	113.39 ± 7.59b	146.10 ± 28.28a	47.11 ± 3.38cd	
7.08	929	Ethanol	MS,KI	ND	783.68 ± 95.68b	724.63 ± 55.38b	772.47 ± 150.73b	1745.35 ± 370.09a	506.51 ± 59.38b	950000^a^
14.26	1222	1-Butanol, 3-methyl-	MS,KI	ND	78.88 ± 1.57a	46.54 ± 3.58b	ND	ND	ND	4^a^
17.57	1371	1-Hexanol	MS,KI	ND	64.39 ± 3.75b	82.55 ± 3.61a	74.58 ± 0.96a	ND	35.77 ± 2.04c	5.7^a^
19.1	1458	1-Octen-3-ol	MS,KI	87.58 ± 6.22	ND	ND	ND	ND	ND	1.5^a^
19.46	1480	1-Heptanol	MS,KI	ND	ND	17.00 ± 1.95	ND	ND	ND	5.5^a^
20.05	1517	2-Ethyl-1-hexanol	MS,KI	ND	ND	ND	ND	ND	7.13 ± 1.77	NF
22.8	1723	1-Non-anol	MS,KI	ND	ND	11.34 ± 1.32	ND	ND	ND	45.5^a^
26.04	2020	Phenylethyl alcohol	MS,KI	ND	136.87 ± 8.85a	74.05 ± 11.49b	148.35 ± 22.73a	134.29 ± 26.71a	49.84 ± 2.76b	564.2^a^
		**Subtotal**		87.58 ± 6.22d	1088.23 ± 103.16b	956.11 ± 62.45b	995.40 ± 176.12b	1879.64 ± 391.06a	629.52 ± 59.91c	
7.81	971	Pentanal	MS,KI	20.46 ± 0.90a	ND	ND	ND	ND	3.38 ± 0.29b	2501.3^a^
10.35	1079	Hexanal	MS,KI	209.11 ± 10.01a	ND	ND	ND	ND	5.98 ± 0.55b	5^a^
11.79	1121	2-Pentenal, (E)-	MS,KI	9.02 ± 1.40	ND	ND	ND	ND	ND	NF
13.1	1175	Heptanal	MS,KI	34.12 ± 3.33	ND	ND	ND	ND	ND	2.9^a^
14.24	1221	2-Hexenal, (E)-	MS,KI	7.72 ± 2.13	ND	ND	ND	ND	ND	17^e^
15.77	1284	Octanal	MS,KI	30.05 ± 1.90	ND	ND	ND	ND	ND	0.6^a^
16.69	1327	2-Heptenal, (Z)-	MS,KI	27.99 ± 1.40	ND	ND	ND	ND	ND	NF
16.87	1336	2-HexeNFl, 2-ethyl-	MS,KI	4.80 ± 2.03	ND	ND	ND	ND	ND	NF
17.96	1391	Non-anal	MS,KI	118.83 ± 16.83a	2.77 ± 0.45b	3.10 ± 0.81b	2.44 ± 0.87b	ND	1.67 ± 0.59b	1.1^a^
19.75	1497	Decanal	MS,KI	4.47 ± 1.34	ND	ND	ND	ND	ND	0.1^e^
19.87	1505	2,4-Heptadienal, (E,E)-	MS,KI	20.09 ± 3.59	ND	ND	ND	ND	ND	15.4^a^
20.38	1540	2-Non-enal, (E)-	MS,KI	19.82 ± 3.52a	1.39 ± 0.76b	0.71 ± 0.10b	ND	ND	0.40 ± 0.10b	0.08^e^
20.41	1542	Benzaldehyde	MS,KI	ND	8.06 ± 2.70d	16.10 ± 2.99c	24.47 ± 4.10b	20.87 ± 18.93b	42.95 ± 4.75a	750.9^a^
21.27	1602	2,4-Octadienal, (E,E)-	MS,KI	9.56 ± 1.39	ND	ND	ND	ND	ND	NF
21.91	1652	2-Decenal, (E)-	MS,KI	19.09 ± 5.00	ND	ND	ND	ND	ND	NF
22.08	1666	Benzeneacetaldehyde	MS,KI	16.05 ± 2.87a	1.54 ± 0.09b	ND	ND	ND	ND	4^e^
22.72	1717	2,4-Non-adienal, (E,E)-	MS,KI	19.39 ± 3.26	ND	ND	ND	ND	ND	0.09^e^
22.92	1734	Benzaldehyde, 3-ethyl-	MS,KI	3.40 ± 0.62a	ND	0.17 ± 0.03b	ND	ND	ND	NF
23.24	1761	2-Undecenal	MS,KI	12.84 ± 3.77a	ND	0.61 ± 0.12b	ND	ND	ND	NF
24.01	1828	2,4-Decadienal, (E,E)-	MS,KI	85.16 ± 32.89a	2.52 ± 0.71b	2.62 ± 0.21b	ND	ND	ND	NF
24.25	1850	Benzaldehyde, 2,5-dimethyl-	MS,KI	5.25 ± 0.29a	2.03 ± 0.16c	3.06 ± 0.53b	1.98 ± 0.19c	ND	1.37 ± 0.06d	NF
27.22	2141	Pentadecanal	MS,KI	ND	1.50 ± 0.27a	ND	1.05 ± 0.18b	1.78 ± 0.35a	0.57 ± 0.08c	NF
		**Subtotal**		677.23 ± 65.68a	19.82 ± 3.58c	26.38 ± 4.45c	29.93 ± 3.83c	22.65 ± 19.27c	52.93 ± 5.99b	
9.79	1057	2,3-Pentanedione	MS,KI	7.50 ± 1.07	ND	ND	ND	ND	ND	5505.6^a^
13.71	1200	2-Heptanone, 4-methyl-	MS,KI	ND	ND	2.47 ± 0.29b	2.28 ± 0.27b	ND	3.12 ± 0.26a	NF
14.87	1247	3-Octanone	MS,KI	ND	ND	2.05 ± 0.23b	ND	ND	6.22 ± 0.13a	21.5^a^
15.68	1281	2-Octanone	MS,KI	ND	3.10 ± 0.53b	3.01 ± 0.27b	3.21 ± 0.61b	ND	5.77 ± 0.43a	50.3^a^
16.1	1298	1-Octen-3-one	MS,KI	12.18 ± 2.15a	0.82 ± 0.16b	1.30 ± 0.09b	1.33 ± 0.14b	ND	ND	NF
16.73	1329	6-Octen-2-one	MS,KI	ND	ND	1.70 ± 0.02b	2.93 ± 0.52a	ND	1.18 ± 0.08b	NF
18.3	1410	3-Octen-2-one	MS,KI	5.39 ± 0.91	ND	ND	ND	ND	ND	NF
23.99	1826	1-Phenyl-2-butanone	MS,KI	ND	0.93 ± 0.01b	0.89 ± 0.10b	0.96 ± 0.25b	1.79 ± 0.27a	0.46 ± 0.07c	NF
26.5	2067	2(3H)-Furanone, dihydro-5-pentyl-	MS,KI	ND	6.32 ± 0.89b	5.58 ± 0.97b	7.44 ± 0.20a	ND	4.08 ± 0.64c	NF
		**Subtotal**		25.07 ± 2.11a	11.17 ± 0.90d	16.99 ± 0.49c	18.15 ± 0.51c	1.79 ± 0.27e	21.95 ± 1.14b	
12.88	1166	Dodecane	MS,KI	ND	7.34 ± 1.31	ND	ND	ND	ND	NF
17.8	1383	Tetradecane	MS,KI	ND	4.82 ± 0.94b	2.71 ± 0.27c	ND	7.83 ± 1.32a	ND	NF
18.78	1439	Undecane, 3,8-dimethyl-	MS,KI	ND	ND	ND	ND	2.70 ± 0.48	ND	NF
19.61	1489	Pentadecane	MS,KI	5.72 ± 1.56a	ND	4.19 ± 0.95b	ND	ND	ND	NF
		**Subtotal**		5.72 ± 1.56b	12.15 ± 2.24a	6.90 ± 1.20b	ND	10.54 ± 1.75a	ND	
14.07	1214	Furan, 2-pentyl-	MS,KI	225.16 ± 40.90a	58.94 ± 8.96b	42.18 ± 5.89bc	27.99 ± 1.91bc	40.81 ± 1.49bc	19.20 ± 6.11c	5.8^a^
15.95	1292	*Trans*-2-(2-Pentenyl)furan	MS,KI	20.98 ± 1.98a	3.12 ± 0.54b	2.50 ± 0.32bc	2.06 ± 0.10bc	ND	1.32 ± 0.32c	NF
20.21	1528	2-n-Octylfuran	MS,KI	ND	3.80 ± 0.54a	2.54 ± 0.61b	ND	ND	ND	NF
		**Subtotal**		246.14 ± 42.88a	65.87 ± 9.10b	47.22 ± 6.78bc	30.06 ± 1.90c	40.81 ± 1.49bc	20.52 ± 6.42c	
18.41	1416	Benzene, 1,3-bis(1,1-dimethylethyl)-	MS,KI	ND	73.88 ± 5.93b	50.74 ± 2.55c	59.47 ± 10.58bc	121.54 ± 19.18a	22.28 ± 4.02d	NF
28.95	2310	2,4-Di-tert-butylphenol	MS,KI	21.38 ± 3.09c	58.75 ± 3.42b	46.56 ± 6.67b	48.61 ± 9.14b	122.22 ± 16.23a	29.95 ± 2.55c	NF
		**Subtotal**		21.38 ± 3.09e	132.63 ± 9.13b	97.30 ± 7.40c	108.08 ± 15.76bc	243.76 ± 33.14a	52.23 ± 5.99d	

*KI, kovats index. S1, inoculated with *Kluyveromyces marxianus* NCYC1425; S2, inoculated with *Torulaspora delbrueckii* Biodiva; S3, inoculated with *Saccharomyces cerevisiae* Lalvin EC-1118; S4, inoculated with *Pichia kluyveri* Frootzen; S5, inoculated with *Candida versatilis* NCYC1433. All fish sauce fermented for 7 days. OT = Odor thresholds: ^*a*^ value obtained from Giri et al. (2010), ^*b*^ value obtained from Niu et al. (2019), ^*c*^ value obtained from Zhu et al. (2018), ^*d*^ value obtained from Zhao et al. (2017), ^*e*^ value obtained from Javed et al. (2018). ^*a*–*e*^ Values in the same row with different letters were significantly different (*P* < 0.05). “ND”: not detected. “NF”: not found. Results: Mean ± SD, *n* = *3*.*

Esters were the major volatile compounds produced during the fermentation, mainly consisting of acetate esters and ethyl esters. S4 presented the highest level of esters (*P. kluyveri* Frootzen, 6621 μg/L), followed by S1 (*K. marxianus*, 1120 μg/L), which were markedly higher than that of S5 (24 μg/L) (*P* < 0.05). In addition, there was no significant difference in ester concentrations between S5 and fish hydrolysate (*P* > 0.05). Furthermore, the largest amounts of ethyl acetate; 1-butanol, 3- methyl-, acetate and acetic acid, 2-phenylethyl ester over 1000 μg/L were also observed in S4.

Volatile acids including acetic acid and medium chain fatty acids were detected in all samples. The highest level of total acids was observed in S4 (147 μg/L), followed by S3 (114 μg/L), while no significant (*P* > 0.05) differences were observed between S1, S2, and S5 (48–63 μg/L). Several kinds of ketones were also detected after fermentation ranging from 2 to 22 μg/L, compared to 26 μg/L of fish hydrolysate.

The total alcohols concentrations of S1–S4 were 1088, 956, 995, and 1880 μg/L, respectively, significantly higher than S5 (630 μg/L) (*P* < 0.05). A lower ethanol level was observed in S5 among the inoculated samples partially due to its lower glucose consumption (Table 1). The aldehydes mainly consisted of aliphatic aldehydes that decreased from 677 to 20–53 μg/L with yeast fermentation. Among them, significantly (*P* < 0.05) lower levels of straight chain aldehydes and unsaturated aldehydes were detected in S1-S4 (2–8 μg/L) than S5 (12 μg/L).

The fermentation with both soy sauce yeast and non-soy sauce yeast led to a significantly (*P* < 0.05) lower level of furans, especially 2-pentylfuran and *trans*-2-(2-pentenyl)furan. Furthermore, a significant increase of alkanes (C12-C15) was observed in S1 and S4 (*P* < 0.05). However, it has been reported that C8-C19 alkanes did not have important effects on flavor because of their high odor threshold values (Xu et al., 2014).

## Discussion

### Yeast Populations and pH Changes During Fermentation

This difference of yeast populations between S1–S4 and S5 was likely caused by the lack of salt in the fish hydrolysate. Fermentation with no salt led to a slower growth rate of *C. versatilis* NCYC 1433, which is a halotolerant strain isolated from fermenting soy sauce. The results indicated that these non-soy sauce yeasts had good growth and survival in an unsalted and acidic environment relative to *C. versatilis* NCYC 1433. This initial pH decline was probably attributed to the release of organic acids (except added lactic acid) produced by yeasts (Figures 3C–G) and dissolved CO_2_.

### Utilization of Sugar and Production of Organic Acids

The reduction of glucose content in all inoculated samples was due to the metabolism of yeasts. All non-soy sauce yeasts had a higher rate of glucose consumption than soy sauce yeast (S5), indicating that non-soy sauce yeasts had a higher fermentative activity in the unsalted system. Normally, glucose is firstly convert into pyruvic acid (Figure 3C) via glycolysis in the cytoplasmic matrix, with the production of citric acid, malic acid and succinic acid by the TCA cycle (Chen and Liu, 2016; Varela, 2016), or converted into ethanol and carbon dioxide via alcoholic fermentation (Lu et al., 2017b). Ethanol is not only an important flavor carrier but also a precursor to ethyl esters, which are important odorants in yeast fermentation (Table 2). It was also reported that high levels of residual sugars could cause a lagging effect on perception of aromas (Villamor et al., 2013). In addition, the heat treatment after 2% (w/v) glucose addition might lead to the Maillard reaction between glucose and protein/peptides/amino acids, resulting in a lower level of glucose (16.85 g/L).

The additional pyruvic acid in S4 was possibly derived from glycerol pyruvate pathway (Ciani et al., 2006). An opposite result was reported by Chen and Liu (2016), who found *S. cerevisiae* Lalvin EC-1118 produced higher amounts of pyruvic acid than *P. kluyveri* Frootzen in lychee wine. This phenomenon could be caused by the different fermentation conditions. Moreover, the reduced pyruvic acid of S4 in the late fermentation could be transformed into acetaldehyde (Cherry et al., 2012). Citric acid (Figure 3D), malic acid (Figure 3E) and succinic acid (Figure 3F) are known to be the critical intermediate products of the TCA cycle. The faster increasing rate of succinic acid observed in S3 was possibly attributed to glyoxylate cycle for succinic acid production in *S. cerevisiae* Lalvin EC-1118 strains in addition to the TCA cycle (Raab and Lang, 2011; Yan et al., 2014). Acetic acid is believed to be produced in yeast by the enzymatic oxidation of acetaldehyde, which is the product of pyruvic acid decarboxylation (Freer, 2002).

### Changes of Free Amino Acids

The interaction between these taste-active amino acids can make a direct contribution to the taste of fish sauce. Free amino acids are not only linked to the taste, but also important aroma precursors. It is reported that valine, leucine and phenylalanine can be catabolized to generate 2-methyl-1-propanol (isobutyl alcohol), 3-methyl-1-butanol (isoamyl alcohol) and phenylethyl alcohol, respectively, via the Ehrlich pathway (Chen and Liu, 2016). These alcohols can be further converted into volatile esters to affect the overall aroma profile by yeasts via esterification or alcoholysis (Gao et al., 2018).

3-Methyl-1-butanol, the metabolic product of leucine, was detected in fish sauce, which is subsequently converted into 1-butanol, 3- methyl-, acetate by yeast (Gao et al., 2018). The leucine concentrations of samples fermented with non-soy sauce yeasts (S1 and S3) were significantly lower than that of soy sauce yeast (*P* < 0.05), with detection of more 3-methyl-1-butanol or 3-methyl-1-butanol, acetate (Table 2). And no 3-methyl-1-butanol and its corresponding ester was found in S5. The results indicated the non-soy sauce yeasts could utilize leucine for aroma substances production.

The valine concentrations in S1, S3, and S4 significantly decreased after fermentation (*P* < 0.05). Similarly, the declined valine might be converted into isobutyl alcohol or its corresponding ester (isobutyl acetate). Actually, isobutyl acetate was only found in S4 and no isobutyl alcohol was detected at the end of fermentation (Table 2). Moreover, phenylethyl alcohol and its related esters (acetic acid, 2-phenylethyl ester and propanoic acid, 2-phenylethyl ester; Table 2) were detected in all samples except S5. The results suggested that the non-soy sauce yeasts could also utilize valine and phenylalanine to generate odorants.

Methionine has been reported as a precursor for distinct odorants of fish sauce such as dimethyl sulfide, dimethyl disulfide and dimethyl trisulfide (Udomsil et al., 2011). However, no sulfur-containing compounds were detected in all samples (Table 2), indicating that these non-soy sauce yeasts could not utilize methionine to produce volatile sulfur-containing compounds in the unsalted and acidic system during a short period.

### Generation of Volatile Compounds

For starting matrix, the aldehydes consisted mostly of straight chain aldehydes and unsaturated aldehydes, which were the metabolites of unsaturated fatty acid, caused by fish endogenous enzymes or autoxidation, especially in heat treatment. Furans could be formed by the Amadori rearrangement pathways (Giri et al., 2010), which was associated with heat treatment.

A higher level of esters in S4 and S1 indicated that *P. kluyveri* Frootzen and *K. marxianus* NCYC1425 had a stronger ester-producing capacity in fish hydrolysate fermentation. More acetate esters and ethyl esters in S4 could be attributed to higher levels of precursors such as acetic acid and ethanol generated by *P. kluyveri* Frootzen, which agree with Lu et al. (2017b). Ester compounds are common volatile components of flavor in food, providing the desirable fruity aroma to the overall aroma profile, due to their low odor thresholds (Zhao et al., 2017; Javed et al., 2018; Zhu et al., 2018; Niu et al., 2019). In this study, ethyl acetate; 1-butanol, 3- methyl-, acetate; decanoic acid, ethyl ester; and acetic acid, pentyl ester (S4); acetic acid, hexyl ester (S4); heptanoic acid, ethyl ester (S3); acetic acid, 2-phenylethyl ester (S4) could contribute to the overall aroma as their concentrations exceed their odor thresholds.

*S. cerevisiae* was reported to have the lipolytical ability to release fatty acids in the previous study (Gao et al., 2016b). It was considered that volatile straight chain fatty acids and ketones were responsible for the cheesy odor of fish sauce (Peralta et al., 1996; Fukami et al., 2002). Meanwhile, S4 and S3 had a significant higher level of fatty acids than other samples (*P* < 0.05). Therefore, *P. kluyveri* Frootzen *and S. cerevisiae* Lalvin EC-1118 had a higher potential to enhance the cheesy odor of fish sauce.

One endogenous alcohol (1-octen-3-ol) was detected in fish hydrolysate, which could be generated from the oxidation of linoleic acid or arachidonic acid (Zhao et al., 2015). A new series of aliphatic alcohols (C2–C9) and phenylethyl alcohol were detected after fermentation. These higher alcohols are produced either from sugars or from amino acids (via Ehrlich mechanism) (Chua et al., 2018). Although present at higher levels, ethanol made a less direct contribution to flavor, due to its relatively high odor threshold (Peralta et al., 1996; Giri et al., 2010). However, it could affect the overall aroma as the precursor of esters as well as aroma compound carrier.

This aldehydes decrease was probably caused by its degradation, reduction to alcohols or further reactions with other compounds, such as amino acids and Maillard other hand, the aerobic respiration of yeast consumed dissolved oxygen and thus inhibited the autoxidation to reduce the production of aldehydes. Normally, straight chain aldehydes generally provide green and grassy aromas, while unsaturated aldehydes are linked with fishy note (Giri et al., 2010). Moreover, significantly (*P* < 0.05) lower levels of straight chain aldehydes and unsaturated aldehydes were detected in S1–S4, indicating wine and dairy yeasts, especially *P. kluyveri* Frootzen, exhibited a variously stronger ability to diminish some certain aldehydes related to distinct odor of fish sauce.

It has been reported that unsaturated aldehydes, pyridines, sulfur-containing compounds and amines contributed cooperatively to the undesirable odor of fish sauce. The volatile compounds detected in this study contained esters, acids, alcohols, aldehydes, ketones, alkanes, furans and aromatics, but not the above-mentioned fish sauce distinct odorants. In addition, different degrees of yeasty aroma could be smelt from the samples, depending on yeast. These findings indicated that the fermentation of non-soy sauce yeasts could effectively diminish the unpleasant smell of fishy, sweaty, rancid and fecal notes, and enrich the favorable flavors at the same time.

### Principal Component Analysis

**FIGURE 4:**
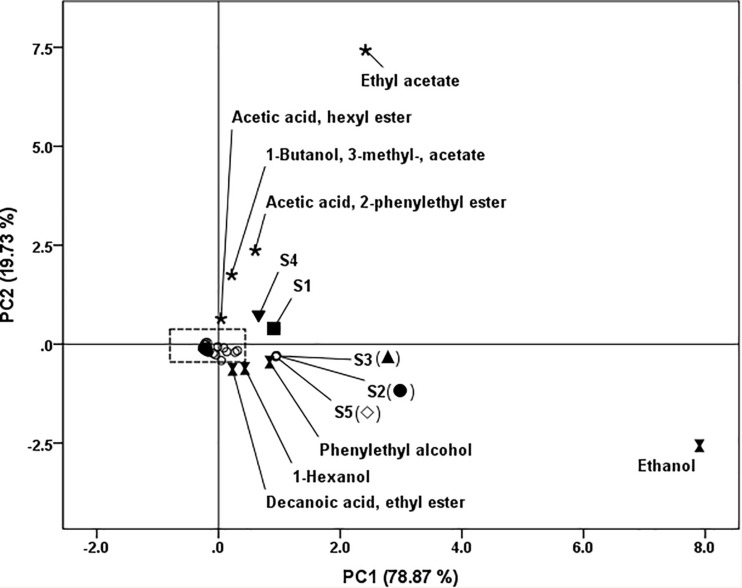
Loadings and scores of principal component analysis of fish sauce fermented with different yeasts. S1 (■): inoculated with *Kluyveromyces marxianus* NCYC1425; S2 (.): inoculated with *Torulaspora delbrueckii* Biodiva; S3 (▲): inoculated with *Saccharomyces cerevisiae* Lalvin EC-1118; S4 (▼): inoculated with *Pichia kluyveri* Frootzen; S5 (◊): inoculated with *Candida versatilis* NCYC1433.

In order to evaluate the aroma profile of fish sauce with different yeasts treatments, principal component analysis (PCA) was conducted using the data of volatiles from Table 2 except fish hydrolysate volatiles. The results are shown in Figure 4. Two principal components were extracted from five variables accounted for 98.60% (PC1 78.87%, PC2 19.73%). The samples were classified into two separate groups. The first group contained S1 and S4; the second group contained S2, S3, and S5. S1 and S4, located at the first quadrant, were correlated with acetate esters (ethyl acetate; 1-butanol, 3- methyl-, acetate; acetic acid, hexyl ester; acetic acid, 2-phenylethyl ester). S2, S3 and S5, located at the fourth quadrant, were related to ethanol, 1-hexanol, 2-phenylethyl alcohol and decanoic acid, ethyl ester. The distribution of volatile compounds in the quadrants provided the information about the differences of wine and dairy yeast.

## Conclusion

This research demonstrated that *T. delbrueckii* Biodiva, *S. cerevisiae* Lalvin EC-1118, *P. kluyveri* Frootzen and *K. marxianus* NCYC1425 had a higher fermentative activity than *C. versatilis* NCYC1433 (a soy sauce yeast) in a fish sauce production from tilapia fish head hydrolysate with salt-free and acidic conditions in a short period. Especially, inoculation of *K. marxianus* NCYC1425 and *P. kluyveri* Frootzen could effectively diminish the distinctive notes of traditional fish sauce, and enrich the fruity note at the same time. It concluded that non-soy sauce yeasts with a high aromatic capacity are suitable for fish sauce flavor modification and to develop a fast fermentation process for salt-free fish sauce from fish head. These applications could increase the acceptability of fish sauce and improve the utilization of fish by-products.

## Data Availability Statement

All datasets generated for this study are included in the manuscript/supplementary files.

## Author Contributions

SL and PG designed the study. SL and WX supervised the study. PG and XL performed the experiments. PG wrote the manuscript. SL reviewed the manuscript.

## Conflict of Interest

The authors declare that the research was conducted in the absence of any commercial or financial relationships that could be construed as a potential conflict of interest.
